# Miswired Enhancer Logic Drives a Cancer of the Muscle Lineage

**DOI:** 10.1016/j.isci.2020.101103

**Published:** 2020-04-29

**Authors:** Berkley E. Gryder, Marco Wachtel, Kenneth Chang, Osama El Demerdash, Nicholas G. Aboreden, Wardah Mohammed, Winston Ewert, Silvia Pomella, Rossella Rota, Jun S. Wei, Young Song, Benjamin Z. Stanton, Beat Schäfer, Christopher R. Vakoc, Javed Khan

**Affiliations:** 1 Genetics Branch, National Cancer Institute, NIH, Bethesda, MD 20892, USA; 2 University Children's Hospital, Zurich, Switzerland; 3 Cold Spring Harbor Laboratory, 1 Bungtown Road, Cold Spring Harbor, NY 11724, USA; 4 Biologic Institute, Redmond, WA, USA; 5 Department of Oncohematology, Ospedale Pediatrico Bambino Gesu' Research Institute, IRCCS, Rome, Italy; 6 Center for Childhood Cancer & Blood Diseases, The Abigail Wexner Research Institute at Nationwide Children's Hospital, Columbus, OH, USA

**Keywords:** Biological Sciences, Chromosome Organization, Molecular Mechanism of Gene Regulation, Cancer

## Abstract

Core regulatory transcription factors (CR TFs) establish enhancers with logical ordering during embryogenesis and development. Here we report that in fusion-positive rhabdomyosarcoma, a cancer of the muscle lineage, the chief oncogene *PAX3-FOXO1* is driven by a translocated *FOXO1* super enhancer (SE) restricted to a late stage of myogenesis. Using chromatin conformation capture techniques, we demonstrate that the extensive *FOXO1 cis*-regulatory domain interacts with *PAX3*. Furthermore, RNA sequencing and chromatin immunoprecipitation sequencing data in tumors bearing rare PAX translocations implicate enhancer miswiring across all fusion-positive tumors. HiChIP of H3K27ac showed connectivity between the *FOXO1* SE, additional intra-domain enhancers, and the *PAX3* promoter. We show that *PAX3-FOXO1* transcription is diminished when this network of enhancers is ablated by CRISPR. Our data reveal a hijacked enhancer network that disrupts the stepwise CR TF logic of normal skeletal muscle development (PAX3 to MYOD to MYOG), replacing it with an “infinite loop” enhancer logic that locks rhabdomyosarcoma in an undifferentiated stage.

## Highlights

• Muscle lineage enhancer logic is miswired in PAX3-FOXO1-driven rhabdomyosarcoma
• PAX7-FOXO1, PAX3-NCOA1, and PAX3-INO80D tumors show evidence of miswired enhancers
• FOXO1 super enhancer is myogenically activated and sustains PAX3-FOXO1 expression
• Oncogenic translocations select for miswired enhancers around disordered proteins

## Graphical Abstract

**Figure:**
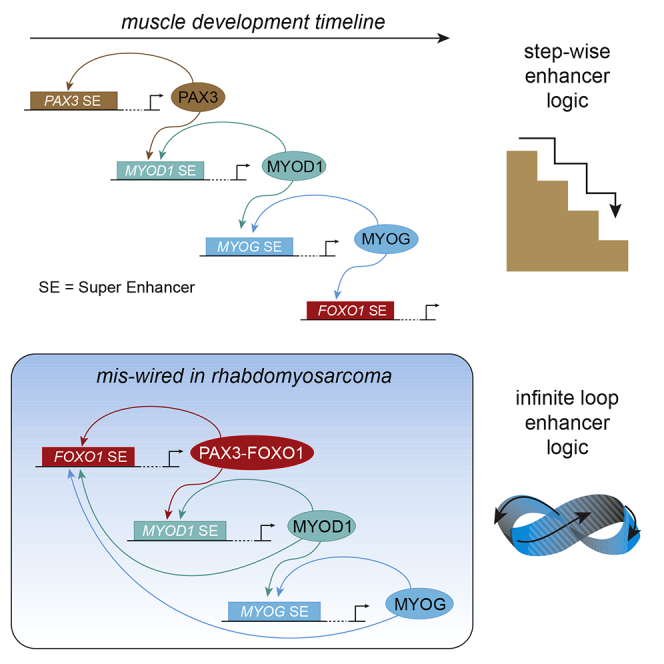

## Introduction

Control of the expression of the core regulatory transcription factors (CR TFs) that guide developmental decision making are directed by logical enhancer elements (Boyer et al., 2005, Chen et al., 2008, Lee and Young, 2013, Vermunt et al., 2019). These genomic elements, when heavily activated, become super enhancers (SEs) with unusually large deposits of active histone marks, chromatin regulators, and transcriptional coactivators (Hnisz et al., 2013). Chromosomal rearrangements allowing SEs to drive oncogene expression is an emerging mechanism in tumor biology (Bandopadhayay et al., 2016, Northcott et al., 2014, Xia and Wei, 2019, Zimmerman et al., 2018). Alveolar (fusion-positive) rhabdomyosarcoma (FP-RMS), an aggressive skeletal muscle cancer of childhood, often possesses chromosomal translocations, involving commonly *PAX3* and *FOXO1* genes, rarely *PAX7*-*FOXO1*, and in exceptional cases *PAX3*-*INO80D* and *PAX3*-*NCOA1* fusions (Shern et al., 2014). Disruption of CR TF transcription is effectual as FP-RMS treatment (Gryder et al., 2017, Gryder et al., 2019a, Gryder et al., 2019b). During normal skeletal muscle development, PAX3 initiates specification of the muscle lineage and is shut off during myogenic differentiation. Consequently, master regulators MYOD and finally MYOG promote muscle progenitor cells to exit cell division and complete muscle differentiation (Hettmer and Wagers, 2010). However, although FP-RMS cells express these master regulators needed to trigger muscle differentiation program, they are halted in an early myoblastic and thus more proliferative state and are not able to complete cell differentiation. Fusion gene products are thought to be responsible for the inability of FP-RMS to differentiate. However, the mechanism of how the oncogenic fusions lock FP-RMS cells in their myoblast state has not been fully understood. In this study, we test the hypothesis that the chromosomal translocation event resulted in novel enhancer/promoter interactions to maintain robust expression of the oncogenic fusion protein in FP-RMS.

Previously, we uncovered a strong dependence on general SE function for tumor survival, with PAX3-FOXO1 being a chief determinant of SE formation in collaboration with MYOD and MYOG (Gryder et al., 2017). Using chromatin conformation capture (3C, 4C-seq, HiChIP) and chromatin immunoprecipitation (ChIP) (ChIP sequencing [ChIP-seq], ChIP-Rx)-based assays, we here study a key SE 300 kb distal to *FOXO1*, and its interconnected smaller enhancer elements, and examine its function in FP-RMS. We propose that hijacking SEs bound by myogenic CR TFs allows for continued expression of oncogenic *PAX* fusions, thus circumventing normal myogenic enhancer logic.

## Results

### Chromosomal Translocation Imports the *FOXO1* Super Enhancer to the *PAX3* Promoter

**Figure 1:**
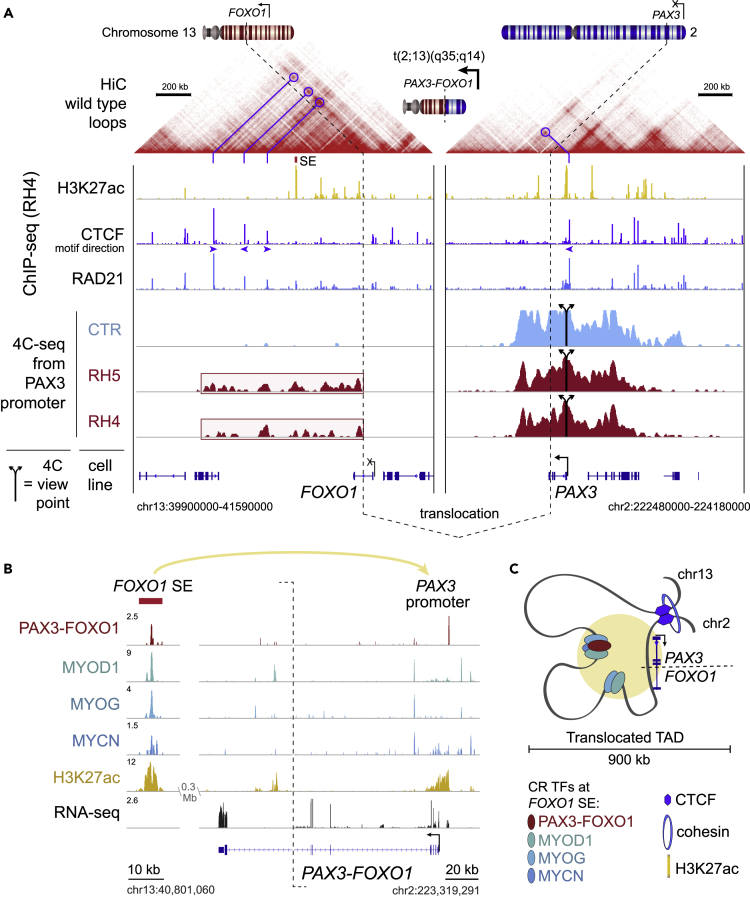
Translocation Structures an Insulated Neighborhood Surrounding PAX3-FOXO1 (A) Wild-type loops indicated by Hi-C profile from human GM12878 cells. ChIP-seq demonstrates binding locations of H3K27ac, CTCF, and RAD21 in RH4 cells. 4C-seq reveals looping between viewpoints at CTCF sites bounding FOXO1 enhancers, and the PAX3 promoter, in translocation-negative (CTR) and translocation-positive (RH5, RH4) cells. Viewpoints are indicated by split arrows, and translocation breakpoints are indicated by dotted lines. (B) ChIP-seq signal for master transcription factors and H3K27ac, and RNA-seq signal, in reads per million (RPM), at the FOXO1 super enhancer (SE) and PAX3-FOXO1 fusion gene, in RH4 cells. (C) Schematic of the translocation creating a new topologically associated domain (TAD) bringing the *PAX3* promoter (chr2) under the control of *FOXO1* SE and other smaller enhancers (chr13).

Precisely how PAX3-FOXO1 locks the cells into a myoblastic state unable to differentiate is unknown. Proper enhancer-promoter interactions are enabled by constraints in 3D chromatin folding, determined by CTCF and cohesin-formed loops at topologically associated domain (TAD) boundaries (Barrington et al., 2019, Dixon et al., 2012, Dowen et al., 2014, Nora et al., 2017). *PAX3* is normally silenced during progression past the myoblast stage of muscle differentiation. PAX3 expression during embryogenesis is tightly controlled, and structural variation that disrupts the PAX3 TAD causes limb malformation (Lupiáñez et al., 2015). We hypothesized that the fusion event results in novel enhancer/promoter looping events to maintain fusion protein expression independent of normal lineage control. Hi-C data (Rao et al., 2014) indicated three candidate topological loops containing wild-type *FOXO1* that exist in normal cells. We found by ChIP-seq that all of these were occupied by RAD21 (of the cohesin complex) and CTCF in FP-RMS RH4 cells (Figure 1A). CTCF-binding events that form loops most often have binding motif sequences that are antiparallel (and point inward toward each other) (Rao et al., 2014). The CTCF motif orientation at the first and third of these sites near *FOXO1* were found to be antiparallel with the CTCF motif near the PAX3 promoter, permissive of chromatin loop formation via extrusion after the translocation.

To identify interacting domains *cis* to the *PAX3* promoter after the translocation, we used circularized chromatin conformation capture followed by sequencing (4C-seq) from viewpoint anchors around the *PAX3* promoter and *FOXO1* genes on chromosomes 2 and 13. Remarkably, looping was detected between the *PAX3* promoter and multiple candidate enhancers downstream of *FOXO1* and was restricted between the intronic fusion breakpoint in *FOXO1* and the predicted topological boundary (Figure 1A). The outermost TAD-boundary looping interaction was confirmed by Sanger sequencing of the 3C PCR product (Figures S1A–S1C). Notably, each of the 3 CTCF sites 3′ of *FOXO1* formed looping interactions with *PAX3* only in translocation-positive RH4, but not in the translocation-negative RMS cell line CTR (Figure S1D). A previous study of 4C-seq in FP-RMS cell lines has shown similar interactions consistent with our results (Vicente-García et al., 2017), but here we provide the first ChIP-seq-informed functional interpretation of these interactions as CTCF-bounded enhancers that contain critical CR TFs. Next, we analyzed this region with genomewide Hi-C contact map data in RH30 cells overlaid ChIP-seq for H3K27ac, confirming interactions between the *FOXO1 cis*-regulome and *PAX3* (Figure S1E) in an independent FP-RMS cell line. We hypothesize that these newly juxtaposed enhancer elements keep the *PAX3* promoter on the translocated allele active. These enhancers could sustain the continual *PAX3-FOXO1* expression and the oncogenic process because they harbor strong binding sites for MYOD1, MYCN, and MYOG (Figures 1B and 1C).

### Rare PAX Fusions Implicate Enhancer Miswiring

**Figure 2:**
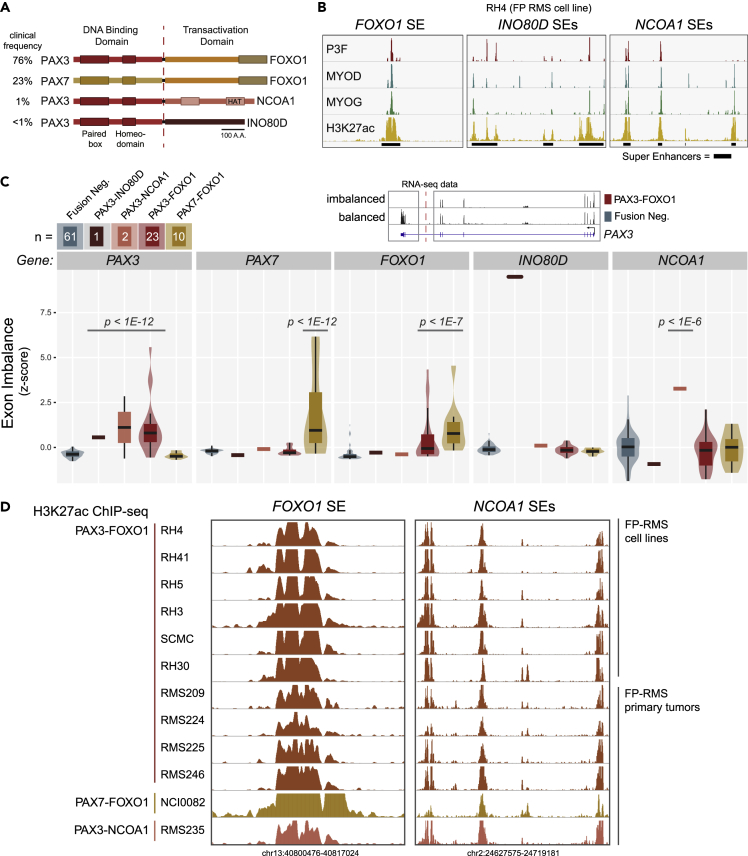
SEs and Allele-Specific Expression at Rare PAX3 Translocation Partners (A) PAX fusions and their clinical frequency in tumors of patients with FP-RMS. (B) SEs in RH4 (PAX3-FOXO1-bearing cells) include not only those near *FOXO1* but also *INO80D* and *NCOA1* in RH4 cells. (C) Exonic imbalance measure in RNA-seq data from primary tumors and cell lines of FN-RMS (n = 61), FP-RMS with PAX3-INO80D fusion (n = 1), PAX3-NCOA1 fusion (n = 2), PAX3-FOXO1 (n = 23), and PAX7-FOXO1 (n = 10). Box plots show the median, quartiles with whiskers at the 1.5 × interquartile range, and distributions plotted as violins. p values were calculated using a two-tailed t test with Welch's correction. Example genome browser of RNA-seq data in FP-RMS is from RH4 and FN-RMS is from SMS-CTR. (D) H3K27ac ChIP-seq in FP-RMS cell lines (n = 6) and primary tumors (n = 6) at the *FOXO1* SE and the *NCOA1* SEs.

Besides the *PAX3-FOXO1* translocation, there are several other PAX translocation variations in FP-RMS including *PAX7-FOXO1*, *PAX3-NCOA1*, and *PAX3-INO80D* (Figure 2A). In each of these cases the 5′ end (N terminal) of *PAX3* or *PAX7* is fused with the 3′ end (C terminal) of *FOXO1* (exons 1–7 [out of 9] of *PAX3* with exons 2–3 of *FOXO1*), *NCOA1* (type 1; exons 1–6 of PAX3 with exons 13–22 of *NCOA1* and type 2; exons 1–7 of *PAX3* with exons 12–22 of *NCOA1*), and *INO80D* (exons 1–7 of *PAX3* fused with exons 9–11 of *INO80D*). There is protein homology between PAX3 and PAX7 (with similar DNA-binding domains), whereas NCOA1 or INO80D do not share any protein homology with the FOXO1 transactivation domain. However, RNA sequencing (RNA-seq) reveals remarkably similar transcriptome profiles from tumors harboring these diverse oncogenic fusions (Shern et al., 2014). In addition, SEs (found in RH4 cells to be bound by FP-RMS-specific CR TFs) exist near all known translocation partner genes (Figure 2B). Therefore, we hypothesized that enhancer miswiring as a result of translocations may be the common theme among all PAX fusion tumors. However, because translocations of *NCOA1* and *INO80D* are rare and cell lines do not exist for them that may allow full epigenomic profiling we reasoned that we could infer epigenetic states (epistates) of the fusion and reciprocal alleles by measuring the exon-level gene expression of fusion partners. We expect that gene fusions involving one active and one inactive gene would result in an exon imbalance, from which we could infer an allele-specific epistate (Figure S2A). In the instance where both alleles have active epistates, we would expect less exonic imbalance (Figure S2B). Thus, from RNA-seq we could infer allele-specific epistates and inform a view of enhancer miswiring in rare forms of FP-RMS.

In FP-RMS with a *PAX3-FOXO1* translocation, if only the promoter of *PAX3* determines the expression of the *PAX3* gene on the wild-type allele and *PAX3-FOXO1* fusion gene on the translocated allele, the expression of all *PAX3* exons will show low variance (as measured by *Z* scoring). The expression of *PAX3* exons will show a high *Z* score if *PAX3-FOXO1* is regulated by the abnormal juxtaposition of the *FOXO1* SE, because the last exons of *PAX3* are not influenced by the *FOXO1* SE (both from the remaining wild-type *PAX3* and the reciprocal *FOXO1-PAX3* translocated allele). Therefore, we examined exon-level expression of the genes involved in translocation using RNA-seq data from tumors of patients with FP-RMS (see Transparent Methods). The RNA-seq data showed that exons before the translocation (3′ or N terminal) are always expressed significantly higher than those beyond the translocation breakpoint (5′ or C terminal) as demonstrated by high *Z* score of exonic expression of *PAX3* or *PAX7* (Figure 2C). This is demonstrated by the high expression of *PAX3* exons 1–7 and low expression of exons 8–9 in the RH4 cell line with *PAX3-FOXO1*, indicating that only the exons involved in the fusion event are expressed due to activation by the *FOXO1* SE. Conversely, exon usage of a gene is balanced in patients lacking the translocation, e.g., *PAX3* in fusion-negative RMS (FN-RMS, SMS-CTR cells, Figure 2C). Importantly, inferring allele selective expression via RNA-seq allows interrogation of extremely rare PAX fusions, *PAX3-INO80D* and *PAX3-NCOA1*. All fusion gene partners showed exonic imbalance (high *Z* scores) resulting from favored expression of the translocated exons (Figures 2C and S2C).

ChIP-seq data in FP-RMS cell lines and patients allowed us to discover recurrent SEs surrounding not only *FOXO1* but also rare partner *NCOA1* (Figure 2D). Although SEs represent only ~4% of enhancers, their unique and consistent presence argues that these diverse fusions may uniformly rewire SEs, which can be active in the epigenomic state of all patients with FP-RMS. Here we report the first epigenomic data generated for a PAX3-NCOA1 patient, and we found that this rare fusion epigenetically phenocopies tumors with PAX3-FOXO1 or PAX7-FOXO1 (Figure 2D). When miswired, these SEs are key elements driving the expression of fusions genes.

### CRISPR Reveals Essentiality of *cis*-regulatory Elements Regulating PAX3-FOXO1

**Figure 3:**
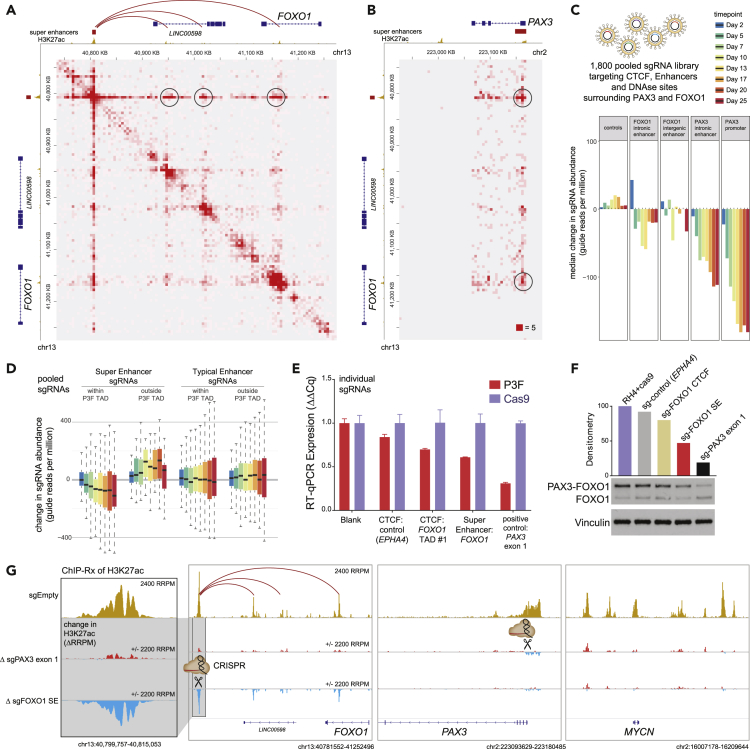
CRISPR Functional Mapping of Non-coding Elements Controlling PAX3-FOXO1 (A) H3K27ac HiChIP reveals structure of FOXO1 SE interactions with smaller intra-TAD enhancer elements (data generated in RH4 cells previously; Gryder et al., 2019a). (B) Interaction by H3K27ac HiChIP between PAX3 promoter and FOXO1 SE and intronic enhancer element. (C) Pooled sgRNA CRISPR screening tiling against *cis*-regulatory genomic elements surrounding *PAX3* and *FOXO1* defined their degree of essentiality. RH4 cells expressing Cas9 were sampled by counting sgRNA abundance using sequencing at the indicated time intervals. (D) Change in sgRNA abundance of pooled CRISPR shows that intra-TAD super enhancers are more critical for RH4 cell survival than typical enhancers or SEs outside TAD boundaries. Data are shown as box plots of the median and first and third quartiles, with whiskers showing 1.5 × interquartile range. (E) Individual sgRNA impact on *PAX3-FOXO1* gene expression after 1 day of transduction in FP-RMS RH4 cells expressing cas9. Bars show median, and error bars represent the SD of technical triplicates. (F) PAX3-FOXO1 protein levels are reduced by individual sgRNAs targeting key *cis*-regulatory elements, especially those targeting the *FOXO1* SE and the first exon of *PAX3*. (G) ChIP-seq with reference exogenous spike-in (ChIP-Rx) for H3K27ac was employed to interrogate the chromatin impact of the sgRNA targeting the *FOXO1* SE. The top track is control ChIP-Rx (sgEmpty), the second track is showing the change (delta RRPM) upon CRISPR of the first exon of *PAX3*, and the third track shows the change in H3K27ac upon CRISPR of the *FOXO1* SE at 24 h post sgRNA transduction. All experiments were performed in RH4 cancer cells.

To build on the evidence from 4C, we set out to gain a more complete dataset confirming the interactions between the enhancer network controlling *PAX3-FOXO1*. Thus, we used HiChIP against H3K27ac to capture protein-directed interaction frequency between acetylated chromatin sites at enhancers and promoters (Gryder et al., 2020, Mumbach et al., 2016). The results identified that the *FOXO1* SE is connected not only to the *PAX3* promoter but also to three smaller intergenic and intronic enhancer elements near or within *FOXO1* (Figures 3A and 3B).

We next measured the contribution of these enhancer elements to the overall survival of FP-RMS cells. We designed a library of single guide RNAs (sgRNAs) against each enhancer or promoter constituent, each DNase hypersensitive site, and each CTCF peak as defined by genome-wide profiles in RH4 cells. We introduced them in a pooled fashion by viral infection into RH4 cells expressing Cas9. The abundance of each sgRNA in the population was then quantified over time using next-generation sequencing (at days 2, 5, 7, 10, 13, 17, 20, and 25). sgRNAs that target the *PAX3* promoter had the strongest impact on RH4 cell viability, as inferred from the largest reduction in guide representation over time (Figure 3C). Among CTCF sites, two candidate anchor sites (*FOXO1*-distal sites #2 and #3) had no negative influence, whereas the outermost CTCF sites (*FOXO1*-TAD boundary site #1 and *PAX3*-TAD boundary) were both reduced by negative selection (Figure S3A). Among TF-bound and H3K27ac-decorated enhancers, sgRNAs targeting SEs within the TAD (the translocation-induced insulated neighborhood containing *PAX3*-*FOXO1*) were more effective than sgRNAs targeting SEs outside this neighborhood, and also more than typical enhancers (Figure 3D).

Individual sgRNAs were next used to study the impact on *PAX3*-*FOXO1* transcription. We confirmed our hypothesis that disruption of the *FOXO1* SE reduced PAX3-FOXO1 at the transcript level and protein level at 24 h after sgRNA infection (Figures 3E and 3F). This resulted in cell growth impairment over time (Figure S3B). To attribute the effect of this sgRNA to direct impairment of the enhancer, we assayed H3K27ac changes by ChIP-Rx (spike in reference normalized ChIP-seq) (Orlando et al., 2014). The results revealed that the *FOXO1* SE was depleted of H3K27ac and that the associated enhancer interaction network (as HiChIP identified) was also drastically reduced of acetylation at the sites interacting with *FOXO1* SE (Figure 3G, see Transparent Methods). Conversely, this enhancer network was not impaired by an sgRNA targeting the first exon of *PAX3*, except for slight reduction in acetylation levels at the *PAX3* promoter (Figure 3G). These data demonstrated that the *FOXO1* SE is essential to maintain the expression of *PAX3-FOXO1* oncogene in RMS.

### *FOXO1* SE Is Activated during a Key Step in Myogenesis

**Figure 4:**
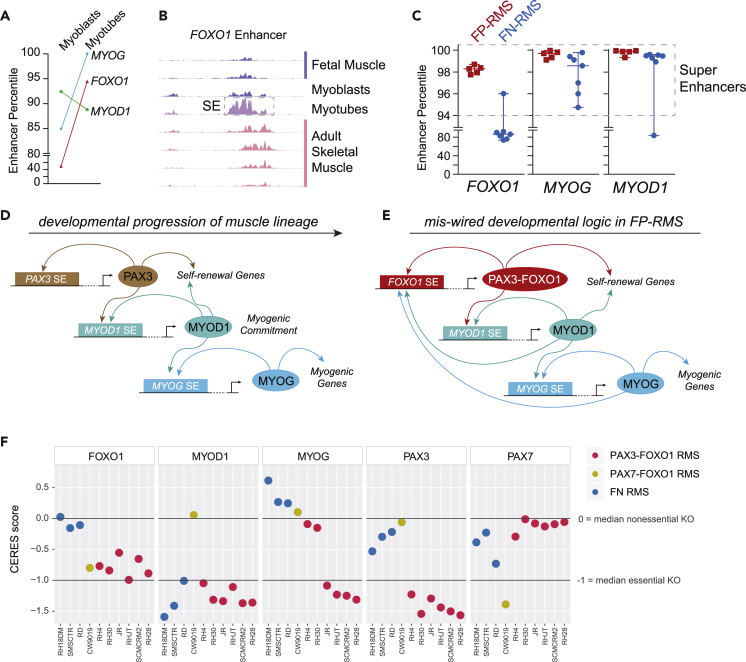
Miswired Super Enhancer Logic Able to Maintain An Oncogenic Cell State (A) Increased FOXO1 and MYOG enhancer rank (by percentile) in the transition from myoblasts to myotubes. (B) H3K27ac signal at the *FOXO1* enhancer in samples from different positions along the muscle lineage development timeline (right). (C) Enhancer percentile for enhancers surrounding *FOXO1*, *MYOG,* and *MYOD1* in RMS, as measured by rank of H3K27ac ChIP-seq signal. Each point shows the enhancer percentile in a different FP- or FN-RMS cell line or primary tumor. (D) Model illustrating normal development of the muscle lineage. (E) Miswiring of myogenic circuitry due to translocation of FOXO1 SE to PAX3 promoter, allowing MYOD and MYOG to activate PAX3-FOXO1. (F) CRISPR dependency data of FOXO1, MYOG, MYOD1, PAX3, and PAX7 in RMS cell lines from the Achilles DepMap project, using CERES scoring scaled to 0 for the median nonessential gene knockout (KO) and −1 for the median essential gene KO.

To examine if the activity of the *FOXO1* SE was coordinated with myogenic steps, we utilized ENCODE data mapping H3K27ac in various stages of the muscle lineage. We found that the *FOXO1* enhancer is transiently transformed into an SE during myogenesis at the same time *MYOG* acquires an SE (Figures 4A and 4B). *FOXO1*, *MYOG*, and *MYOD1* have more highly ranked SEs in FP-RMS when compared with FN-RMS (Figure 4C), in agreement with the notion that FP status is more advance toward myotubes and FN status is more similar in the earlier myoblast state. *MYOG* activation is commonly prevented by mutant RAS signaling through MEK/ERK in FN-RMS tumors, which can be rapidly released via small molecule inhibitors of MEK/ERK (Yohe et al., 2018). Using this system, we asked if the *FOXO1* SE and concomitant *FOXO1* expression was induced alongside *MYOG* activation. Indeed, we not only found *FOXO1* to be upregulated but also observed MYOG invasion on the same SE that is recruited during the PAX3-FOXO1 translocation event in FP-RMS (Figure S4A).

Together, our data suggested that the myogenic lineage timeline (Figure 4D) is miswired in rhabdomyosarcoma to maintain early TFs (granting the self-renewal capacity afforded by the de-differentiated cell state) despite the presence of late terminal differentiation factors (prominently MYOD and MYOG, Figure 4E). This implies that the infinite loop logic could be broken by removal of MYOD and MYOG from RMS cells, and although these TFs are not directly druggable as yet, DepMap CRISPR data strongly suggests that these (and the infinite loop they support) are essential for PAX3-FOXO1-driven RMS growth (Figure 4F).

## Discussion

Two factors can be selected for in rearrangement-driven cancers: oncogenic biochemical function in the case of a resulting fusion protein (Kadoch and Crabtree, 2013) and aberrant expression levels of a proto-oncogene (such as *MYC* or *GFI1*) via enhancer hijacking (Northcott et al., 2014). In FP-RMS, PAX fusions are selected for both an oncogenic fusion protein product and miswired enhancer logic, effectively reprograming core regulatory TF networks. Lacking a translocation, the FN-RMS subtype has aberrations in signaling pathways that enable circumvention of normal myogenic logic (Slemmons et al., 2017, Yohe et al., 2018).

PAX3 activates MYOD by binding and activating *MYOD1* SEs, but then shuts off presumably because MYOD does not work backward to upregulate *PAX3* (MYOD ChIP-seq shows no binding in the *PAX3* promoter, unlike the *MYOG* promoter, Figure S4B). Lacking enhancers responsive to MYOD/MYOG, the remaining wild-type alleles of *PAX3/7* in FP-RMS tumors are silent. Our data suggest that newly juxtaposed enhancer elements initiate and continually drive *PAX3-FOXO1* expression, implicating that enhancer miswiring is at the heart of the oncogenic process in FP-RMS. When the *FOXO1* SE is translocated to regulate *PAX3*, late myogenic factors (MYOG/MYOD) work through this SE to drive an early myogenic factor (PAX3), changing a “progressive” enhancer logic into an “infinite loop” enhancer logic. Similarly, CR TF logic circuits are self-reinforcing in self-renewal and proliferative states such as embryonic stem cells (Whyte et al., 2013).

Analysis of RNA-seq for patients with non-FOXO1 partners (INO80D, NCOA1) of PAX3 reveals a remarkably similar transcriptome (Shern et al., 2014), despite not being TFs themselves, and having no sequence homology to the activation domain (AD) of FOXO1. It has been shown that TFs can perform their function even when their ADs are swapped with those of other TFs (Hope and Struhl, 1986). This tolerance to diverse AD sequence may be explained by the fact they share the common property of being intrinsically disordered, a feature needed to support phase separation capacity of TFs (Boija et al., 2018). Indeed, the portions of FOXO1, INO80D, and NCOA1 involved in PAX3 fusion oncoproteins are predicted to be heavily disordered (Figures S5A–S5C). Remarkably, although transcription factors as a class are heavily disordered (Figure S5D), PAX fusion partners are particularly disordered (similar to FET family fusions like EWSR1). A related partner MAML3 is almost entirely disordered (Figure S5E), and *PAX3-MAML3* fusions occur in biphenotypic sinonasal sarcoma (SNS) but not in RMS (Wang et al., 2014). *MAML3* lacks an SE in myogenesis or RMS, and our model would therefore predict the absence of *PAX3-MAML3* translocations in RMS. SNS may arise from a cell of origin whose epigenome has a lineage-restricted enhancer at MAML3, which gets recruited in SNS tumorigenesis. We believe that even these non-TF fusion partners are actually acting as TFs by inducing liquid-liquid phase condensate formation and likely serving as a scaffold for the intrinsically disordered domains of proteins (i.e., Mediator, BRD4, and RNA Pol2) that are essential for PAX3 fusions to drive transcription from distal enhancers.

Many disordered proteins in the genome are not involved in *PAX3* fusions. A parallel criterion for a successful tumorigenic fusion could be the presence of an active SE in the same lineage step as *PAX3* (such as those SEs proximal to *FOXO1*, *INO80D,* and *NCOA1* in the RMS-specific epigenetic state). SE-containing loci may be enriched in translocations for two reasons. First, active enhancers are transcriptionally active and early replicating (Siefert et al., 2017), and thus likely more susceptible to double-strand breakage (Barlow et al., 2013). Second, among translocations which form, those resulting in overexpression of an oncogene are selected for, and SEs can enable such continued overexpression. The appearance of certain SEs is transient and logically restricted to certain points in development and thus may be restricting the potential miswiring events that could give rise to an “infinite loop” in CR TF logic. We propose that this can explain, at least in part, the selection of translocation partners in FP-RMS tumors and provides a paradigm likely relevant to other translocation-driven cancers.

### Limitations of the Study

RNA-seq and ChIP-seq analyses used for inferring enhancer miswiring in rare PAX3/7 fusion tumors is limited as we have only validated it functionally in cell lines bearing the most common fusion PAX3-FOXO1. Furthermore, the bioinformatic analysis and predictions we have made regarding the intrinsically disordered regions of FOXO1, INO80D, and NCOA1, have not been validated with experiments to show liquid droplet formation. Last, the enhancer data for myogenesis are limited to only a few steps along this lineage, according to what is publicly available. These limitations leave important work to be pursued in future work by the field.

### Resource Availability

#### Lead Contact

Javed Khan (khanjav@nih.gov).

#### Materials Availability

Plasmids, cell lines are available upon request. Primary tumor samples are unavailble for public distribution but please contact us if interested in collaborative exploration with these rare materials.

#### Data and Code Availability

Newly generated ChIP-seq data from primary RMS tumors has been made publicly available through the Gene Expression Omnibus (https://www.ncbi.nlm.nih.gov/geo/). The GEO accession number is GSE136799. Code is available at https://github.com/GryderArt.

## Methods

All methods can be found in the accompanying Transparent Methods supplemental file.

## Supplemental Information

Document S1. Transparent Methods and Figures S1–S5
