# Supplementary material for: Miswired Enhancer Logic Drives a Cancer of the Muscle Lineage
Source: iScience. 2020 Apr 29;23(5):101103. doi: 10.1016/j.isci.2020.101103 (PMC7226896; doi:10.1016/j.isci.2020.101103)
Supplement: Document S1. Transparent Methods and Figures S1–S5 [file mmc1.pdf]

iScience, Volume *23*

Supplemental Information

Miswired Enhancer Logic Drives a Cancer of the Muscle Lineage

Berkley E. Gryder, Marco Wachtel, Kenneth Chang, Osama El Demerdash, Nicholas G. Aboreden, Wardah Mohammed, Winston Ewert, Silvia Pomella, Rossella Rota, Jun S. Wei, Young Song, Benjamin Z. Stanton, Beat Schäfer, Christopher R. Vakoc, and Javed Khan

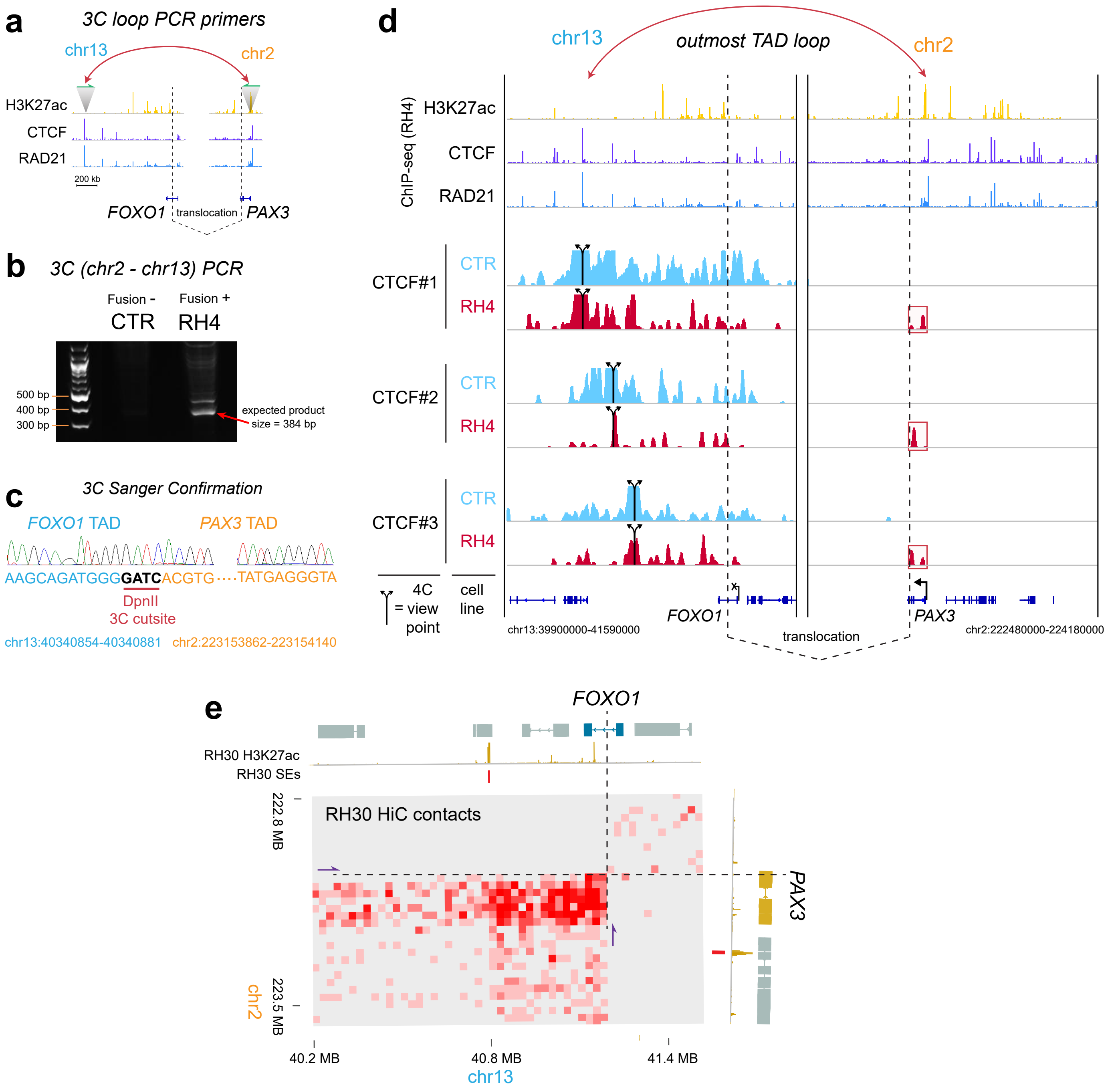

Figure S1, related to Figure 1.

a. Diagram of 3C confirmation PCR primer placement

b. 3C confirmation PCR in fusion negative (CTR) and fusion positive (RH4) cells.

c. Sanger validation of 3C PCR product.

d. 4C-seq of CTCF sites: 3 viewpoint sites distal to *FOXO1* on chromosome 13, which show interaction with chromatin proximal to the *PAX3* promoter and CTCF anchor.

e. PAX3-FOXO1 translocation induced HiC contacts between the FOXO1 cis-regulome and the PAX3 gene body/promoter in RH30. H3K27ac ChIP-seq from RH30 also shown, with SEs for the same cell line.

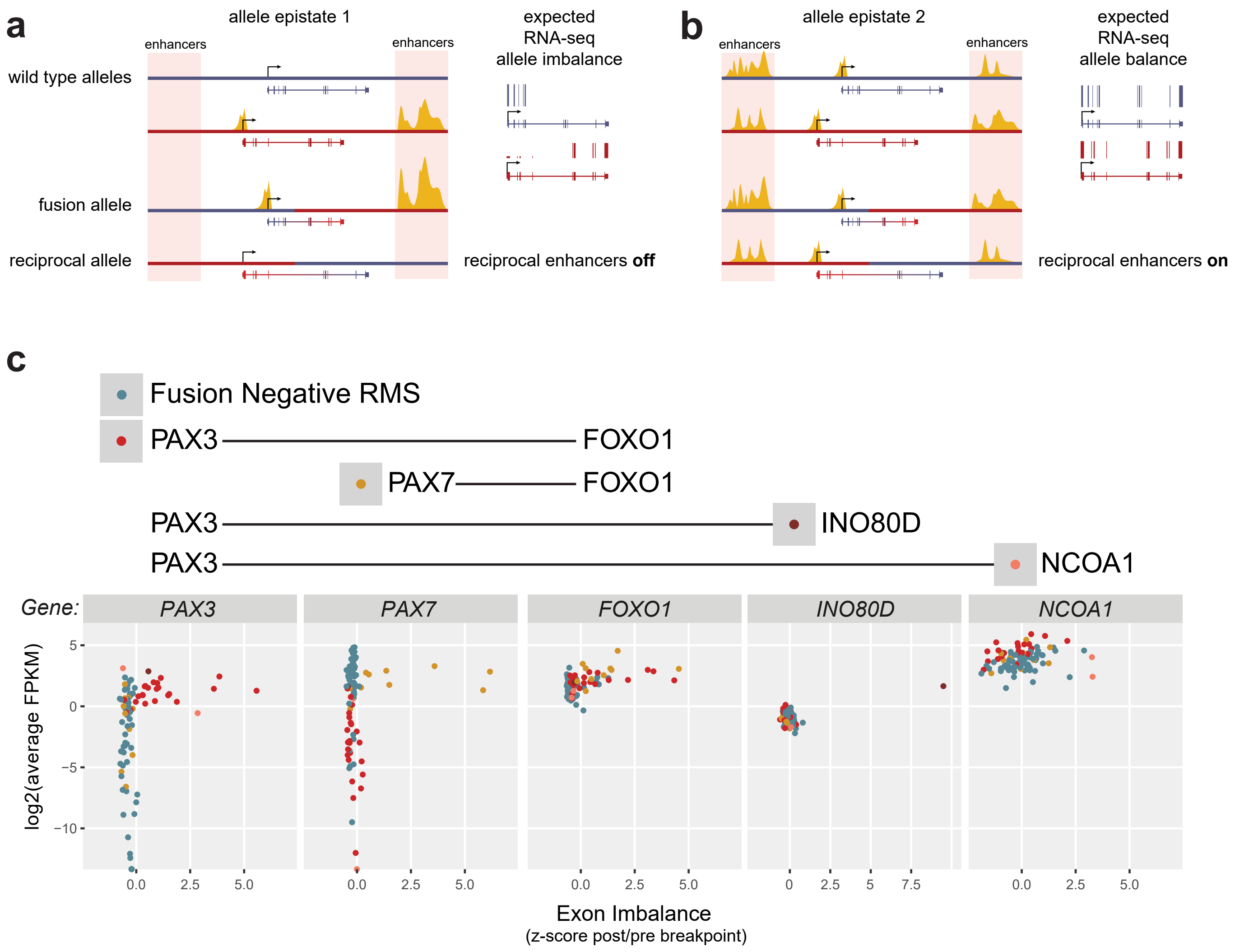

Figure S2, related to Figure 2.

a. Allele specific epigenetic state (epistate) is expected to create an exonic imbalance by activating fused allele and not reciprocal allele lacking active enhancers.

b. If both partners of a fusion gene are surrounded by active enhancers then the wild type, fusion and reciprocal alleles being active would result in a more balanced exon level expression than in (a).

c. Exon imbalance for PAX fusion partner genes, plotted against RNA expression levels.

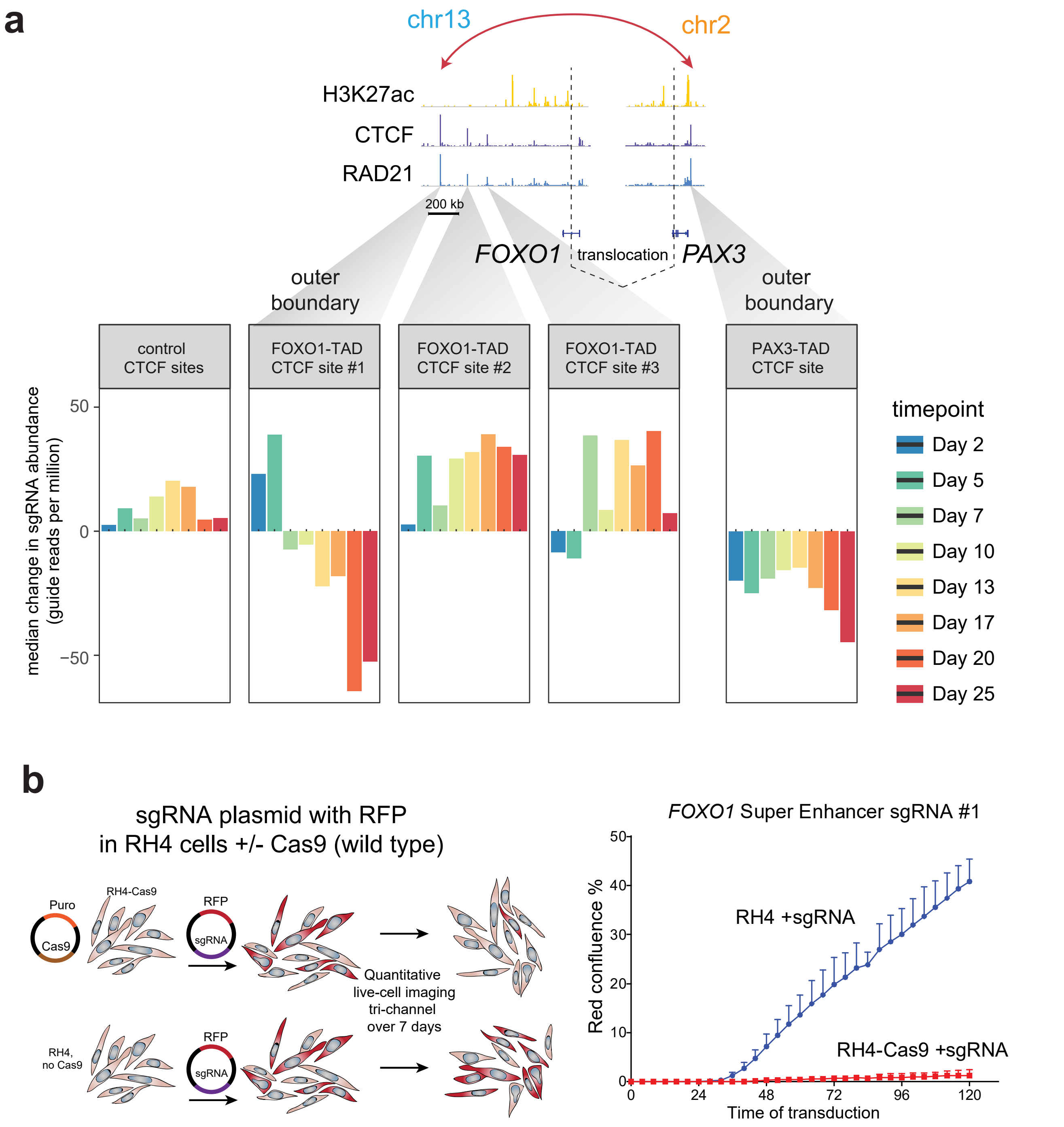

Figure S3, related to Figure 3.

a. Pooled sgRNA CRISPR interrogation of CTCF sites surrounding *PAX3* and *FOXO1* reveals importance of the two most distal sites, but not two intervening CTCF bound locations distal to *FOXO1*.

b. Time course Cas9-negative selection test with sgRNA targeting FOXO1 SE and Red Fluorescent Protein (RFP) expression, with and without Cas9 in RH4 cells.

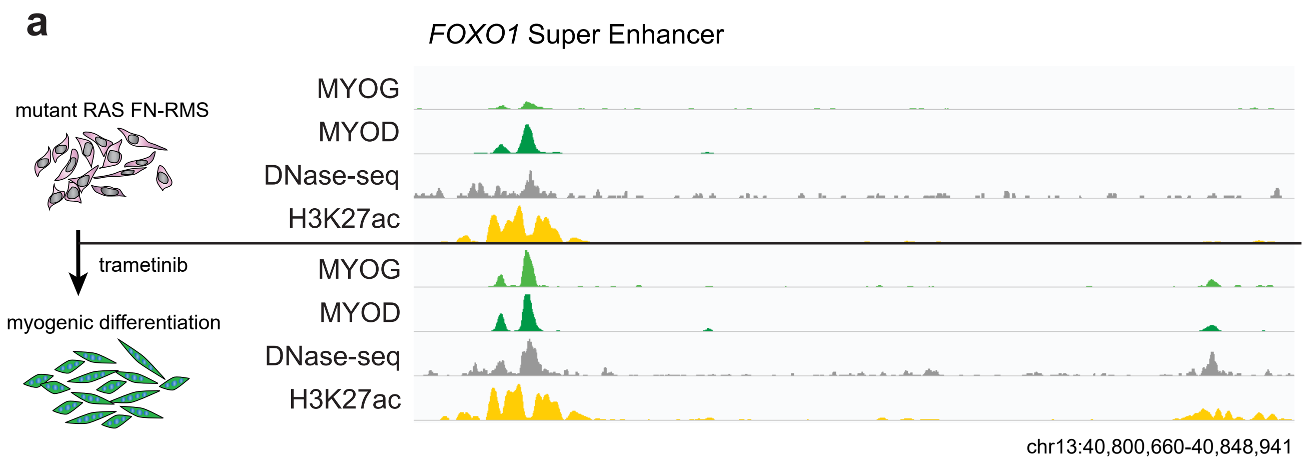

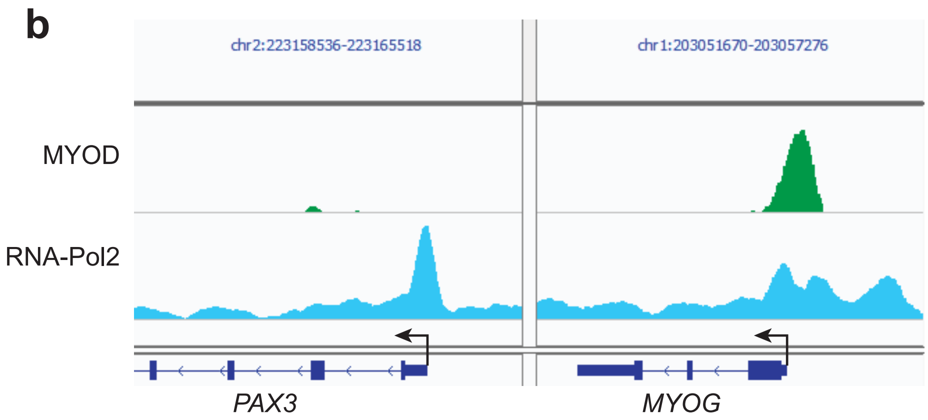

Figure S4, related to Figure 4.

a. MYOG invades the *FOXO1* super enhancer during trametinib-induced myogenesis in SMS-CTR cells, a PAX-fusion lacking cell line driven by mutant HRAS (Q61K).

b. MYOD ChIP-seq alongside RNA Pol2 ChIP-seq at the *PAX3* and *MYOG* promoters in RH4 cells (*PAX3-FOXO1* translocated FP-RMS cancer cells)

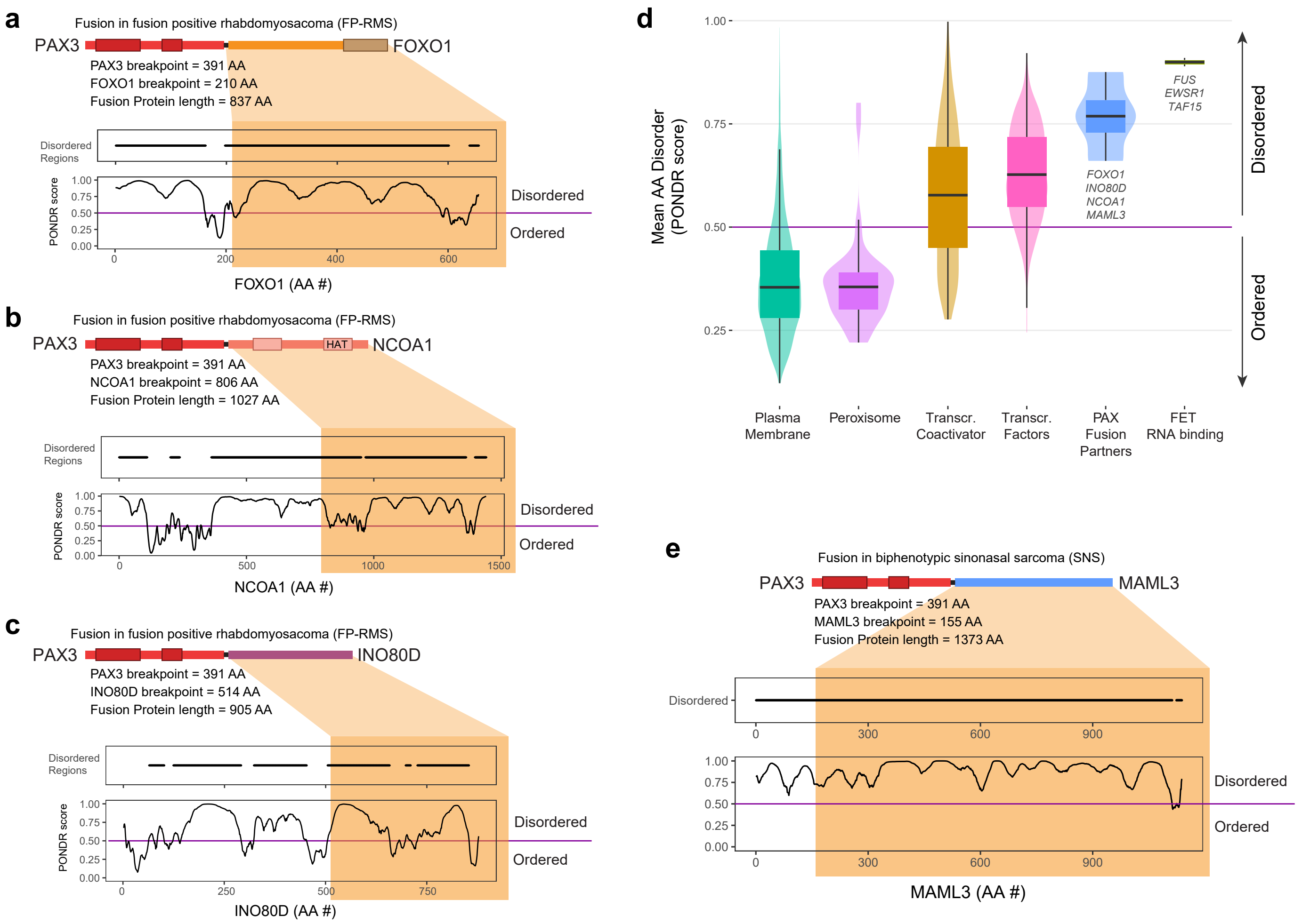

Figure S5, related to Figure 4.

a. PAX3-FOXO1 fusion gene, with PONDR (Predictor of Natural Disordered Regions) score (http://www.pondr.com/) and disordered regions mapped for FOXO1 amino acids.

b. PAX3-NCOA1 fusion gene, with PONDR score for NCOA1.

c. PAX3-INO80D fusion gene, with PONDR score for INO80D

d. Distribution of average disorder PONR scores among protein families, including transcriptional coactivators and transcription factors, alongside PAX3 fusions and FET family fusion partners.

e. PAX3-MAML3 fusion gene, with PONDR score for MAML3.

Transparent Methods

Circularized chromatin conformation capture (4C-seq)

4C-seq was performed on RH4, RH5 and CTR cells as previously described (Gryder et al., 2017). Briefly, cells were grown in DMEM at 37 °C, then chemically crosslinked with 1% formaldehyde for 12 minutes. Then, *in-situ* digestion and *in-situ* re-ligation of 3D contacts (keeping nuclei intact) was performed using DpnII as the 4-bp DNA cutter. Following re-ligation, Csp6I was used to reduce template size, followed by relegation to circularize for inverse PCR at viewpoints of interest surrounding *PAX3* and *FOXO1*. 4C samples of RMS cell lines were amplified using bait region primers for PAX3-promoter (F: CAAGGAGTCCTGGTGCCAAA, R: CACTGGCCGGTGAGAAGG). We also studied three CTCF sites on the FOXO1 side with the following primers: FOXO1-CTCF.1 (F: GCTCCCACAGAAGAAGCAGA, R: GGTGGAGACAGAGGCAGTAC), FOXO1-CTCF.2 (F: CACACACAAGCAAGCACAGA, R: AGCCTCATTACCACTTTTGAACA), and FOXO1-CTCF.3 (F: TCAGGAAGGTTCAAACTTACTTTCC, R: CACGCACGCATAAAAGAGCA). Illumina TruSeq ChIP Library Prep Kit was used on purified inverse PCR products, and sequenced (75 bp single end) with an Illumina NextSeq500 (and, are typically multiplexed with ChIP-seq experiments to increase sequence diversity, which is needed because most reads from a single 4C viewpoint are identical duplicated products from self-ligation).

4C-seq data analysis

Reads from 4C experiments were first filtered to keep only reads containing the bait primer sequence (from the viewpoint), tolerating 1 mismatched base pair. Then, barcodes and viewpoint sequence were trimmed, followed by mapping to hg19 with bwa. Reads surrounding the viewpoint (within 4 kb) were removed to aid visualization of informative distal contacts. Smoothing was performed by averaging over a sliding window of 5 kb and visualized in IGV.

3C confirmation PCR and sequencing

Primers were designed to validate interaction between the outermost CTCF boundaries surrounding *PAX3* and *FOXO1* (Extended Data Fig. 1). The left primer overlapped a digestion site detected to form a long-range ligation at high frequency in 4C data, with the sequence: AAGCAGATGGG**GATC**ACGTG (DpnII cutsite in bold). The right primer was designed close to the PAX3 CTCF site, but before any additional cutsites: GCAGCCAGTGAGATAAGACATTA. PCR was performed using Phusion High Fidelity Master Mix (NEB) with 30 pmol of each primer and 70 ng of 3C DNA template (same template from which 4C was prepared, prior to the second Csp6I cut), with 25 cycles (94 °C for 15 seconds, 55 °C for 30 seconds, 70 °C for 30 seconds) with completed with 1 minute at 70 °C. PCR product was purified with PCR QiaQuick kit (Qiagen), then Sanger sequencing was performed at the Center for Cancer Research Sequencing Facility (CCR-SF).

Exonic imbalance analysis of RMS RNA-seq

RNA-seq read counts were generating using cufflinks at the exon level for each sample (RMS primary tumor data) (Shern et al., 2014). Then, for each RMS tumor (n = 97), the exonic balance before and after the known break points were calculated (average FPKM of pre-breakpoint exons/average FPKM of post-breakpoint exons) for all known translocation partners (*PAX3*, *PAX7*, *FOXO1*, *NCOA1*,

INO80D). Isoforms used for analysis were *PAX3*: NM_181458; *PAX7*: NM_001135254; *FOXO1*: NM_002015; *NCOA1*: NM_147223; *INO80D*: NM_017759
Normal exonic bias was corrected for by taking a z-score across all samples (including FN-RMS samples). Plots of exon imbalance where made in R using ggplot2.

HiC and HiChIP data analysis
HiC data for high-resolution contact frequencies in GM12878 cells (Rao et al., 2014) was downloaded and visualized through the Juicebox desktop application (Durand et al., 2016). Candidate native loop contacts (purple circles, Fig. 1) were annotated manually from visual inspection, and CTCF motif orientation was derived by taking the intersect of CTCF peaks in RH4 cells called with MACS2 (previously published, GEO accession number GSM2214099) and CTCF recognition sequences annotated in the HOMER known motifs collection (http://homer.ucsd.edu/homer/motif/genomeWideMotifScan.html). HiC data in RH30 cells was obtained from ENCODE3.

H3K27ac HiChIP data analysis of data generated in RH4 cells (Gryder et al., 2019) was performed using the Hi-Pro pipeline (Gryder et al., 2020; Servant et al., 2015). We kept only high-quality paired-end tags (PETs), using the HiC-Pro pipeline to filter out reads lacking the restriction site (DpnII), filter out duplicates, and filter out poorly mapped reads. Valid contact pairs remaining were converted to .hic format using the hicpro2juicebox.sh script (https://github.com/nservant/HiC-Pro/tree/master/bin/utils), to enable compatibility with the Juicebox visualization tool.

Lentivirus production
A lentivirus plasmid mix was made by combining Lenti-Rev, Lenti-PM2, Lenti-Tat and Lenti-Vsv-G plasmids at a ratio of 1:1:1:2. HEK293T cells were seeded at 50-60% confluency (2 million cells) in a 10 cm dish in 10 mL media (DMEM, 10% FBS) and incubated at 37°C, 5% CO_2. The next day, 5 µg of transfer plasmid, 10 µg of lentivirus plasmid mix and 36 µL of X-tremeGENE HP DNA transfection reagent (Sigma) were mixed into 1.2 mL serum-free OptiMEM (Gibco), vortexed and incubated for 15 minutes at RT and added to the cells. Media was changed 24 hours after transfection. Viral supernatant was harvested the following three days and filtered through a 0.45 µm syringe filter. Each collection was diluted with 5X PEG-it Virus Precipitation Solution (System Biosciences) and stored at 4°C until the final collection. After the final collection, viral supernatants were centrifuged at 1500g for 30 minutes at 4°C. Viral pellets were resuspended in PBS, aliquoted and stored at -80°C. All transductions were conducted with 8 µg/mL polybrene.

Pooled CRISPR Library Design and Construction
Sequences of enhancer regions (~100 sites) surrounding *PAX3-FOXO1*, CTCF, and SE peaks were derived such that all possible sgRNA guides for *S.pyogenes* Cas9 can be designed within those regions. A total of 1830 sgRNAs were produced. Guides were selected based on having an off-target score of 1.0 denoting the maximum possible score for guides not having any off-target. Oligonucleotides were synthesized as a pool commercially (Twist Biosciences) and then PCR cloned into BsmBI-cut sgRNA expression vector, LRG2.1T, by using gibson assembly. Deep sequencing analysis was performed on Illumina platform and verified that all of sgRNA designs were cloned (data not shown).

Pooled CRISPR-Cas9 screening

The lentiviral sgRNA library was produced as described above. RH4.Cas cells were first transduced with varying concentrations of the pooled lentiviral library to determine the amount needed for a MOI of ~0.3. RH4.Cas cells were then seeded in three 15 cm dishes at 8 million cells per dish. 24 hours later, cells were transduced with the lentiviral library at a low MOI, leading to ~27% positive cells (as determined by FACS analysis of GFP expression) and ensuring over 1000x representation of the library. 48 hours after transduction, virus-containing media was removed and replaced with fresh media. 40 million cells were collected and pelleted at days 2, 5, 7, 10, 13, 17, 20 and 25 post-transduction and 12 million cells were plated at each timepoint for subsequent cell sampling. Coverage at cell level was kept above 1000x throughout the screen.

Sequencing libraries were constructed essentially as previously described (Shi et al., 2015). Genomic DNA (gDNA) from cell pellets was extracted using the AllPrep kit (Qiagen). 32 parallel PCR reactions were performed to amplify sgRNA sequences from gDNA harvested at each timepoint. The total volume of each PCR reaction was 50 µL containing 400 ng of gDNA template, 0.2 µM forward (TCTTGTGGAAAGGACGAAACACCG) and reverse (TCTACTATTCTTTCCCCTGCACTGT) primers, and 25 µL Platinum PCR SuperMix (Thermo Fisher). PCR cycles were: 1x (98°C, 2min) 35x (98°C, 8s, 67°C, 12s 72°C, 10s), 1x (72°C, 5min). Parallel PCR products were pooled together and purified with the QiaQuick kit (Qiagen). After production of sgRNA amplicons from gDNAs by PCR, ends of fragments were repaired using T4 DNA polymerase (NEB), DNA polymerase I large fragment (Klenow) (NEB), and T4 polynucleotide kinase (NEB), followed by addition of 3' A-overhang with Klenow (3'-5'-exo-) (NEB). Amplicons from each sample were ligated with a unique barcode adaptor (pool) and purified using AMPure magnetic beads (Bechman Coulter). Samples were deep sequenced on Illumina platform. Read counts for each sample were ascertained by mapping raw reads to the sgRNA library sequences. Read counts of each sample were normalized to facilitate data analysis.

Construction of individual sgRNA expression plasmids

sgRNA sequences were designed using the CRISPR design tool from MIT (crispr.mit.edu). Pairs of DNA oligonucleotides encoding the protospacer sequences were annealed together to create double-stranded DNA fragments with 4-bp overhangs. These fragments were ligated into BsmBI digested Shuttle_sg_RFP657 plasmid. Plasmid constructs were confirmed via sanger sequencing using the LKO1_5 primer (GACTATCATATGCTTACCGT) on the U6 promoter.

DepMap Achilles Data Analysis

CRISPR-cas9 screening for essential genes was performed by the Broad Institute's DepMap and Achilles team (Meyers et al., 2017), and the data was downloaded from the 2020 Q1 release (https://depmap.org/portal/download/all/) and was processed using custom R scripts (https://github.com/GryderArt/CRISPRtoolkit/).

Supplementary References

Durand, N.C., Robinson, J.T., Shamim, M.S., Machol, I., Mesirov, J.P., Lander, E.S., and Aiden, E.L. (2016). Juicebox Provides a Visualization System for Hi-C Contact Maps with Unlimited Zoom. Cell Systems *3*, 99-101.

Gryder, B.E., Khan, J., and Stanton, B.Z. (2020). Measurement of differential chromatin interactions with absolute quantification of architecture (AQuA-HiChIP). Nature Protocols.

Gryder, B.E., Pomella, S., Sayers, C., Wu, X.S., Song, Y., Chiarella, A.M., Bagchi, S., Chou, H.-C., Sinniah, R.S., Walton, A.*, et al.* (2019). Histone hyperacetylation disrupts core gene regulatory architecture in rhabdomyosarcoma. Nature Genetics *51*, 1714-1722.

Gryder, B.E., Yohe, M.E., Chou, H.-C., Zhang, X., Marques, J., Wachtel, M., Schaefer, B., Sen, N., Song, Y.K., Gualtieri, A.*, et al.* (2017). PAX3-FOXO1 Establishes Myogenic Super Enhancers and Confers BET Bromodomain Vulnerability. Cancer Discovery.

Meyers, R.M., Bryan, J.G., McFarland, J.M., Weir, B.A., Sizemore, A.E., Xu, H., Dharia, N.V., Montgomery, P.G., Cowley, G.S., and Pantel, S. (2017). Computational correction of copy number effect improves specificity of CRISPR–Cas9 essentiality screens in cancer cells. Nature genetics *49*, 1779.

Rao, Suhas S.P., Huntley, Miriam H., Durand, Neva C., Stamenova, Elena K., Bochkov, Ivan D., Robinson, James T., Sanborn, Adrian L., Machol, I., Omer, Arina D., Lander, Eric S.*, et al.* (2014). A 3D Map of the Human Genome at Kilobase Resolution Reveals Principles of Chromatin Looping. Cell *159*, 1665-1680.

Servant, N., Varoquaux, N., Lajoie, B.R., Viara, E., Chen, C.-J., Vert, J.-P., Heard, E., Dekker, J., and Barillot, E. (2015). HiC-Pro: an optimized and flexible pipeline for Hi-C data processing. Genome Biology *16*, 259.

Shern, J.F., Chen, L., Chmielecki, J., Wei, J.S., Patidar, R., Rosenberg, M., Ambrogio, L., Auclair, D., Wang, J., Song, Y.K.*, et al.* (2014). Comprehensive Genomic Analysis of Rhabdomyosarcoma Reveals a Landscape of Alterations Affecting a Common Genetic Axis in Fusion-Positive and Fusion-Negative Tumors. Cancer Discovery.
